# Editorial: The Role of Ceramides in Diabetes and Cardiovascular Disease

**DOI:** 10.3389/fendo.2021.667885

**Published:** 2021-03-12

**Authors:** Scott A. Summers

**Affiliations:** Department of Nutrition and Integrative Physiology, University of Utah, Salt Lake City, UT, United States

**Keywords:** ceramides, lipotoxicity, sphingolipids, diabetes, heart disease

## Introduction

The lipotoxicity hypothesis, first described by the late Roger Unger nearly 30 years ago (1, 2), posits that the excessive delivery of fatty acids to the heart, vasculature, liver, muscle, pancreas and adipose tissue gives rise to the tissue damage that underlies diabetes and cardiovascular disease. Under lipotoxic conditions, the quantity of fatty acids delivered to these tissues exceeds their energy needs and their storage capacity, leading to the aberrant formation of lipid metabolites that impair tissue function. This Research Topic of *Frontiers in Endocrinology* evaluates the role of ceramides and its metabolites in the tissue damage that drives these cardiometabolic disorders.

Ceramides are products of a biosynthetic pathway that adds fatty acids to a sphingoid scaffold derived from palmitoyl-CoA and serine (3). Quantitatively, ceramides and other sphingolipids are minor constituents of the cellular lipidome; they are present at far lower levels than the glycerolipids that comprise the bulk of lipid droplets and cellular membranes (4). The ceramides presumably accumulate when the glycerolipid pathway is saturated, and the residual fatty acids become available as substrates for the enzymes that produce sphingolipids.

**Figure 1:**
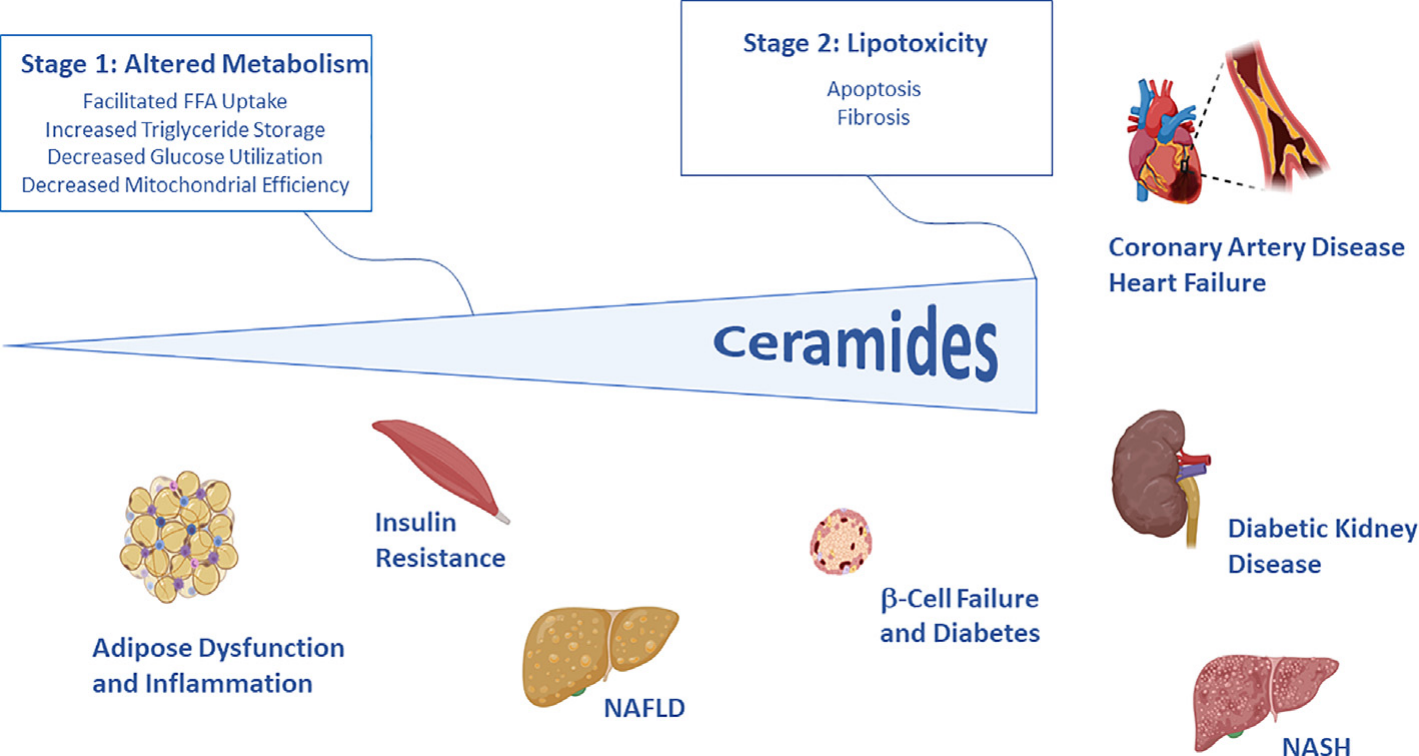
Schematic depicting the two stages of ceramide-driven lipotoxicity. In initial stages, ceramides serve as signals of lipid excess that alter metabolic programs, enabling storage of fat and decreasing utilization of glucose. In the latter stages, ceramides trigger apoptosis and fibrosis, protecting the organism from compromised cells. These cellular events give rise to the key features of diabetes and cardiovascular disease. Image generated using Biorender.

In the first article of the series, entitled ***“Too Much of a Good Thing? An Evolutionary Theory to Explain the Role of Ceramides in NAFLD,”*** Poss and Summers present a conceptual framework that explains the evolutionary basis of the ceramide actions that elicit tissue dysfunction. We speculate that—at an early point in evolution—ceramides conferred upon cells and organisms a survival advantage by protecting membranes from detergent-like fatty acids. They do this by altering membrane properties and by changing metabolic programs; these adaptations facilitate the uptake and storage of fatty acids while decreasing utilization of glucose (**Figure 1**). As ceramide levels increase further, they induce apoptosis and fibrosis, which helps protect organisms from fragile cells that have become compromised by fatty acid overload. While these actions may provide a short-term advantage, the chronic changes in metabolism (e.g., decreased glucose utilization and increased fat deposition) elicit the insulin resistance and dyslipidemia that are early signs of disease progression. Moreover, the increased susceptibility to cell death elicits the terminal organ damage that drives cardiometabolic disease. This set of conserved actions would thus explain many of the key features of the diseases associated with dyslipidemia and obesity.

The other articles in this series provide evidentiary support for this hypothesis, describing in careful and thoughtful detail the means by which ceramides alter tissue function. Moreover, they highlight the numerous instances where inhibition of the enzymes required for ceramide biosynthesis ameliorate diabetes and heart disease.

In the article ***“Sphingolipid Metabolism and Signaling in Skeletal Muscle: From Physiology to Physiopathology,”*** Tan-Chen et al. discuss the ceramide actions that decrease insulin-stimulated glucose utilization in muscle. They also review the numerous interventional studies in rodents that reveal that lowering ceramides ameliorates insulin resistance.

In “***Ceramides in Adipose Tissue,”*** Li et al. discuss the studies indicating that ceramides inhibit glucose uptake, enhance lipid storage, and decrease mitochondrial efficiency of the adipose depots. Interestingly, they also note that ceramides inhibit phosphorylation of hormone-sensitive lipase, providing another mechanism by which ceramides work to lower cellular FFA levels.

In “***Sphingolipids in the Heart: From Cradle to Grave***,” Kovilakath et al. discuss the contribution of sphingolipids to myocardial lipotoxicity and coronary artery disease, two of the key pathogenic features of major adverse cardiac events. The authors describe the key studies indicating that ceramides have deleterious actions on heart function, while its downstream metabolite sphingosine 1-phosphate is cardioprotective.

In ***“The Role of Ceramides in Diabetes and Cardiovascular Disease Regulation of Ceramides by Adipokines,”*** Field et al. review how the broad swath of anti-diabetic and cardioprotective actions of adiponectin—including its prevention of apoptosis of cardiomyocytes and pancreatic β-cells—result from the acceleration of ceramide degradation. Remarkably, they and others have found that the adiponectin receptors are ceramidases that are activated by ligand binding (4, 5).

Beyond these mechanistic studies on the tissue-specific roles of ceramides, additional articles discuss the potential therapeutic value of these studies on ceramides.

In their article ***“Ceramides and Ceramide Scores: Clinical Applications for Cardiometabolic Risk Stratification,”*** Hilvo et al. discuss the numerous studies showing that clinical risk scores based on serum ceramides demarcate patients that are at risk for cardiometabolic disorders.

Lastly, Raichur discusses work on ceramide-targeted therapies, concluding that “***Ceramide Synthases Are Attractive Drug Targets for Treating Metabolic Diseases*.”**

Collectively, these studies describe the exciting body of evidence indicating that ceramides are drivers of diabetes and heart disease. They delineate the novel mechanisms by which ceramides influence metabolism and increase susceptibility to apoptosis and fibrosis. Moreover, they reveal the exciting potential of therapeutic strategies that block ceramide accumulation, which could emerge as treatments for a wide range of cardiometabolic pathologies.

## Author Contributions

The author confirms being the sole contributor of this work and has approved it for publication.

## Conflict of Interest

SS is a co-founder of Centaurus Therapeutics.
